# Krabbe Disease Associated With Mitochondrial Dysfunction in a Chinese Family

**DOI:** 10.3389/fneur.2021.750095

**Published:** 2021-12-16

**Authors:** Liwen Wu, Xiangfu Liao, Sai Yang, Siyi Gan

**Affiliations:** ^1^Department of Neurology, Hunan Children's Hospital, Changsha, China; ^2^The First People's Hospital of Yue Yang, Yueyang, China

**Keywords:** development delay, leukoencephalopathy, NDUFAF1 gene, GALC gene, mitochondria dysfunction

## Abstract

**Background:** Krabbe disease is caused by biallelic mutations of *GALC* gene. *NDUFAF1* gene mutations are related to mitochondrial encephalopathy. To date, there has been no report on the co-pathogenesis of these two gene mutations. There were three children in a family who presented with global developmental retardation. MRI showed lesions in the white matter and dentate nucleus of the cerebellum.

**Methods:** Clinical data of the proband and her family members were gathered in a retrospective manner. Karyotype, FISH, whole exome sequencing was performed using genomic DNAs extracted from peripheral blood samples. Enzyme activities of galactosylceramidase (GALC) and mitochondria were determined to verify gene functions.

**Results:** This study reported a pedigree of leukoencephalopathy, in which 3 of the 4 children showed phenotypes of developmental delay, hearing/visual impairment, and peripheral neuropathy. Mutations of *NDUFAF1* (c.278A>G; p. His93Arg, c.247G> A; p. Asp83Asn) and *GALC* (c.599C>A; p.Ser200^*^) were identified in all three cases. The proband's parents carried these mutations as a heterozygous state. Clinical features, MRI changes, enzyme activity of GALC, and mitochondrial function analysis demonstrated that this pedigree was caused by GALC and NDUFAF1 gene mutations working together.

**Conclusion:** We first report a pedigree of Krabbe disease with biallelic mitochondrial gene *NDUFAF1* mutations. For multiple gene mutations found in genetic testing, clinical phenotypes, gene functions, and family history should be comprehensively analyzed. Gene panel examination may miss pathogenic mutations, and prenatal diagnosis of patients with polygenic inheritance needs careful consideration.

## Background

The development and wide application of genomics and molecular biology have led to the early diagnosis of an increasing number of hereditary diseases but has also raised many important issues. It has been clinically experienced that we may miss some pathogenic mutations because of monitoring and technology errors, or simultaneously detect mutations in several pathogenic genes in a patient, which makes it difficult to determine whether a disease is caused by a single gene or multiple genes and further complicates prenatal diagnosis.

Krabbe disease (KD; globular leukodystrophy globoid; OMIM 245200) is a rare autosomal recessive hereditary disease in which there is lack of galactosylceramidase (GALC) enzyme, which hydrolyzes galactosylceramide and galactosylsphingosine (psychosine, PSY). Most common MRI abnormalities involve deep white matter, dentate nucleus, and cerebellar white matter, with relative sparing of U-shaped fibers under the cortex. However, MRI changes in white matter were more extensive in the cases described in this study compared to those in patients with KD. Few cases of leukoencephalopathy caused by the gene mutation of NDUFAF1 have been reported. Our study extends the clinical and molecular phenotypes of KD and further contributes to identify the common pathogenicity of double gene mutations in this family.

## Methods

### Patient

Three patients (cases 1, 2, and 3) and their healthy 5-year-old brother were included in the study. The three patients were treated in Xiangya Hospital of Central South University, the First People's Hospital of Yue Yang, and Hunan Children's Hospital. Finally, they were managed at the Department of Neurology, Hunan Children's Hospital. The parents of the patients gave written informed consent. This study was approved by the Medical Ethics Committee of Hunan Children's Hospital.

### Whole Exome Sequencing

Exome data were analyzed with a bioinformatics pipeline. The chip IDTxGen Exome Research Panel v1.0 was used to capture the entire gene exons. The capture process was as follows: a total amount of 2 μg genomic DNA was used as input material for DNA sample preparation. Sequencing libraries were generated using xGen Exome Research Panel probes (IDT, United States) and following the manufacturer's recommendations. The sequencing libraries were processed with an Illumina NovaSeq 6000 (Illumina, San Diego, CA, United States) sequencing platform. The sequencing data were evaluated with Illumina Sequence Control Software (Illumina, San Diego, CA, United States) for data reading and bioinformatics analysis. Paired-end clean reads were mapped to the human reference genome (GRCh38/hg38) by BWA, and variations were called *via* GATK and annotated with InterVar. All variants with a minor allele frequency of ≤0.01 in public databases (ExAc, gnomAD, and 1000g) were obtained. Pathogenecity for the candidate variants were evaluated according to the guidelines of American College of Medical Genetics and Genomics (ACMG). Sanger sequencing was performed to verify the mutations.

### JC-1 Metabolic Staining and Flow Cytometry Analysis

We measured the mitochondrial membrane potential (ΔΨm) of peripheral blood to evaluate mitochondrial function. The specimens were stained with JC-1. Briefly, cells were stained with a 500-μL 1 × JC-1 reagent working solution at 37°C for 15 min in 5% CO_2_. The cells were centrifuged for 5 min at 400 × g and were resuspended in phosphate-buffered saline (PBS). Subsequently, the cells were analyzed using an FC 500 flow cytometer (CytekDxp Athena; Cytek Biosciences, Fremont, CA, United States). Mitochondrion-containing red JC-1 aggregates in healthy cells were detectable at λ excitation/λ emission = 585/590 nm, and green JC-1 monomers in apoptotic cells were detectable at λ excitation/λ emission = 510/527 nm.

### Galactocerebrosidase (GALC) Enzyme Activity

A method for determination of the GALC enzyme was established according to references (1). The substrate 6-hexadecanoylamino-4-methylumbelliferyl-beta-d-galactoside (HMU-β Ga1) was purchased from Moscerdam (Shanghai Kanglang Biotechnology Co., Ltd). The substrate buffer was 0.2 mol/L phosphate/0.1 mol/L citric acid (pH 5.2). Four milliliters of EDTA anticoagulant peripheral blood was taken. Leukocytes were separated by dextran method. Cell homogenate was prepared by ultrasonic crushing, and protein concentration was determined by the BCA method. Ten picoliters of white blood cell homogenate (15 ug protein) was mixed with 0.4 mmol/L HMU-β Ga1 and incubated at 37°C for 17 h. Sodium bicarbonate 0.5 mol/L/0.5 sodium carbonate buffer (pH 10.7) was added to terminate the reaction. Fluorescence intensity of free HMU was measured using a Bio-Tek FLx 800 fluorescence analyzer with an excitation wavelength of 350 nm and emission wavelength of 460 nm. Enzyme activity was calculated using HMU as the standard, expressed in nmol/(mg·17 h). When the activity of GALC was significantly lower than that in the normal control group (lower than 10% of the mean), we re-sampled and repeated the test once for confirmation.

## Results

### Case 1

**Figure 1:**
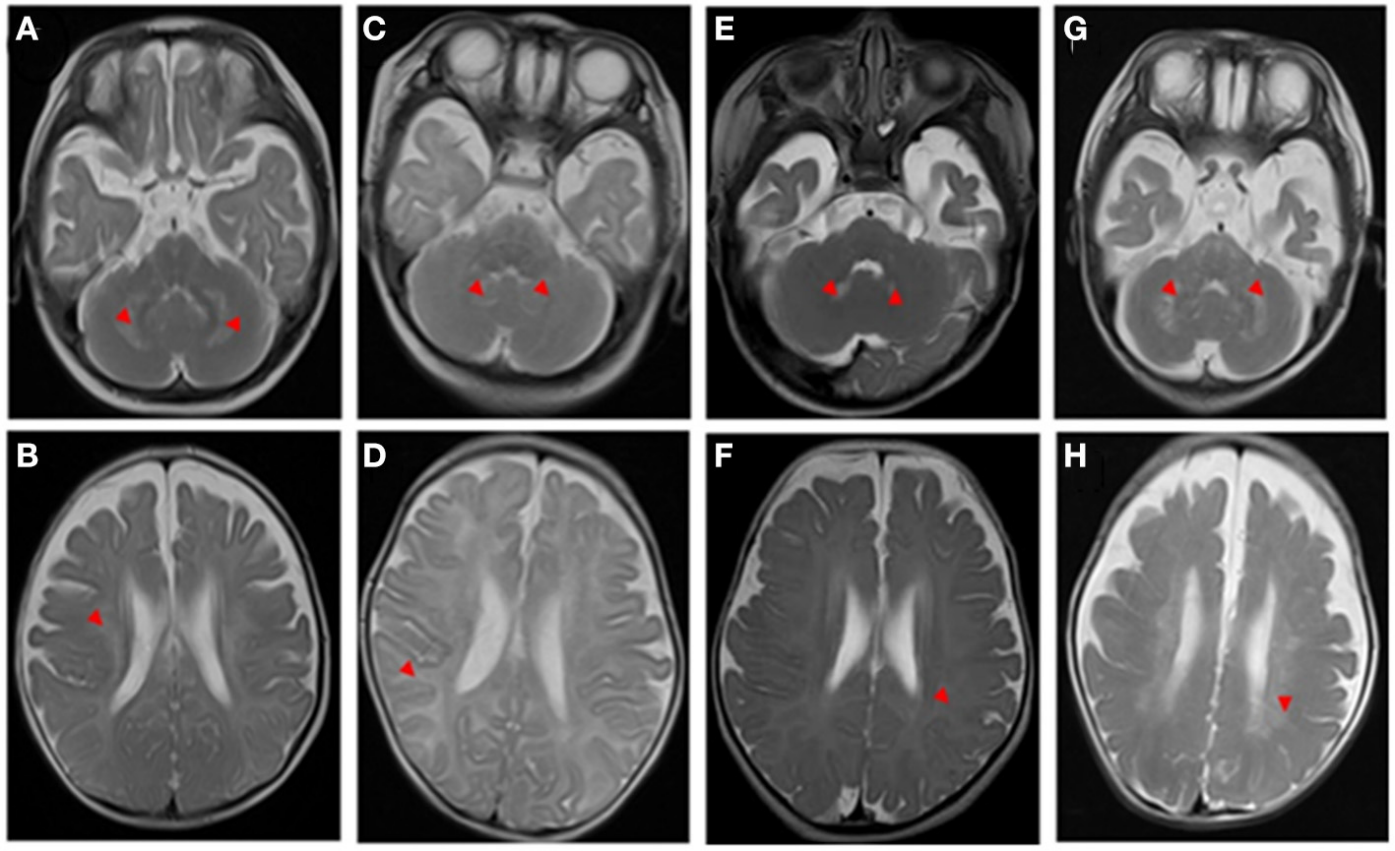
T2-weighted brain magnetic resonance imaging (MRI) of the patients. The red arrowheads show the lesions. Neuroimaging findings (**A,B**, case 1)at the 5-month-old show a cerebellar dentate nucleus and white matter were damaged, and the subcortical U-shaped fibers were significantly involved; (**C,D**, case 2) at a 3-month-old show diffuse lesions in white matter, obvious subcortical involvement of U-shaped fibers, and suspicious lesions in cerebellar dentate nuclei; (**E, F**, case 3) at a 3-month-old show suspicious lesions in the cerebellar dentate nucleus and the change of cerebral white matter is not prominent; **(G,H)** at the 6-month-old evident lesions in the cerebellar dentate nucleus.

A 5-month-old boy who presented with developmental delay and intellectual disability exhibited intractable fever after birth. On physical examination, he had high muscle tension and hyperreflexia of the limbs. Laboratory examination showed that lactate and creatine kinase levels were high. Transverse T2-weighted brain magnetic resonance imaging (MRI) showed abnormally high signal intensity in the cerebellar dentate nucleus, cerebral white matter, and subcortical U-shaped fibers (Figures 1A,B). A nerve conduction study (NCS) revealed moderate peripheral demyelinating sensorimotor neuropathy and visual evoked potential (VEP) and brainstem auditory evoked potential (BAEP) abnormalities. At 10 months of age, his appetite became continually decreased until feeding difficulties developed, and his mental state was bad, as he almost did not have a reaction. Finally, he was regretfully declared dead. The results showed that the child had compound heterozygous mutations (c.278A4G; c.247G4A) in exon 2 in the *NDUFAF1* gene, and that both his parents carried a heterozygous mutation. *NDUFAF1* mutations are considered to be responsible for leukodystrophy (Table 1).

**Table 1:** Clinical characteristics of three cases in the family.

**Patient**	**Case 1**	**Case 2**	**Case 3**
Sex	Male	Female	Female
Onset year	5 months	3 months	3 months
Death age	10 months	6 months	6 months
Symptom	Feeding difficulties Pulmonary infection Axial hypotonia Developmental delay	Seizures Developmental delay	Developmental delay Temperature instability
Physical examination	High muscle tension Limb hyperreflexia	Paroxysmal muscle tone increases	Muscle tone increases Deep reflexes diminished or absent
Blood biochemistry	High lactate and creatine kinase	Lactate and creatine kinase	High ALT/AST/AKP; low GALC enzyme activity (1.1nmol/17 h/mg)
NCV (tibial nerve)/BAEP/VEP	17 m/s (Demyelination changes) BAEP/VEP abnormal	17 m/s (Demyelination changes) BAEP abnormal	19 m/s (Demyelination changes) BAEP abnormal
T2-weighted brain MRI	Cerebellar dentate nucleus and white matter were damaged	Lesions in white matter, U-shaped fibers, cerebellar dentate nuclei	Lesions in cerebral white matter
Variant	*NDUFAF1* (c.278A>G;p. His93Arg c.247G>A; p. Asp83Asn) *GALC* (c.[599C>A]; [599C>A]; p.Ser200*)	*GALC* (c.[599C>A]; [599C>A]; p.Ser200*) *NDUFAF1* (c.278A>G; p. His93Arg c.247G>A; p. Asp83Asn)	*GALC* (c.[599C>A]; [599C>A]; p.Ser200*) *NDUFAF1* (c.278A>G; p. His93Arg c.247G>A; p. Asp83Asn)

### Case 2

Similarly, one of the siblings from birth to 3 months had unsatisfactory development, excessive crying, hearing problems, and paroxysmal muscle tone increases. She was admitted to the pediatric intensive care unit (PICU) of our hospital because of recurrent epilepsy, and it was difficult to control her seizures. The brainstem auditory evoked potential (BAEP) revealed that she had damage to the auditory nerve. Additionally, both motor and sensory nerve conduction velocities were significantly decreased with normal compound action potentials, suggestive of demyelination. Blood tandem mass spectrometry and urinary organic acid analysis displayed normal results. Transverse T2-weighted images showed hyperintensity of white matter, obvious involvement of subcortical U-shaped fibers, and suspicious increased T2 signal in cerebellar dentate nuclei (Figures 1C,D). When she was 6 months old, her persistent high fever worsened with lung infection. She required tracheal intubation-assisted ventilation for a month, her reactions worsened, and she lacked autonomous activities. Ultimately, the parents abandoned treatment, and she was discharged from the hospital. Whole exome sequencing (WES) results showed a homozygous mutation in the *GALC* gene (c.[599C>A]; [599C>A]; p.Ser200^*^). The variant was observed in both parents as a heterozygous state. The homozygous mutation was interpreted as pathogenic according to the ACMG guidelines (Table 1).

### Case 3

Then, another 3-month-old girl in the family suffered from the same problem, which attracted the attention of the parents. It was noticed in early infancy, and she could not perform eye pursuit movements, cooing, social smile, and head control. Simultaneously, the child presented with episodes of fever and pulmonary infection. She had no dysmorphic features. Neurological examination of the patient revealed discomfort crying and a remarkable increase in muscle tone. The results of cranial nerve examination were normal. Her tendon reflexes were diminished or absent. She did not show Babinski signs.

Laboratory examination showed that alanine aminotransferase (ALT), aspartate aminotransferase (AST), and alkaline phosphatase (AKP) levels were 61.3 (0–40), 75.2 (0–42), and 207.1 (34–160) U/L, respectively. All of the following biochemical investigations were unremarkable: acylcarnitine profile, ammonia, lactic acid, creatine kinase, urine amino acids, urine organic acids, coagulation profile, and lipid profile. Brain MRI scans at 3 months demonstrated a suspiciously high T2 signal in the cerebellar dentate nuclei and no obvious changes in the cerebral white matter signal (Figures 1E,F), and a follow-up study at 6 months revealed increased T2 signals in the dentate nuclei and cerebral white matter (Figures 1G,H). Moreover, motor nerve conduction velocities were markedly decreased, indicating peripheral nerve demyelination. The BAEP was recorded, and wave I with very prolonged latency was detected; the I-III, III-V, and I-V interpeak latencies (IPLs) were normal, and there was a suspicious extension of the bilateral BAEP hearing-threshold testing. The VEPs were also abnormal. At 6 months of age, the parents began to notice new symptoms, such as feeding trouble, lack of eye contact, and minimal spontaneous movement, and mental response gradually worsened. She tragically died early in 3 months.

Two probands (cases 1 and 2) of this family had similar symptoms, and it was strange that genetic testing revealed mutations in *NDUFAF1* and *GALC*, respectively. Therefore, Sanger sequencing of these two genes was performed for this patient, and the results showed a homozygous mutation in the *GALC* gene (c.[599C>A]; [599C>A]; p.Ser200^*^) and the two mutations (c.278A>G; p. His93Arg and c.247G>A; p. Asp83Asn) in the *NDUFAF1* gene (Table 1).

It is puzzling that the sequencing of case 1 only detected mutations in the *NDUFAF1* gene (Case 1 was reported in 2013 {Wu L, Leukodystrophy associated with mitochondrial complex I deficiency due to a novel mutation in the NDUFAF1 gene. Mitochondrial DNA A DNA Mapp Seq Anal. 2016; 27(2):1034-7.}), whereas cases 2 and 3 showed mutations in the *GALC* gene. It raises the question which mutations in the two different genes account for nearly identical clinical phenotypes in the family? Then, we checked the original data from these children again and found that case 1 also carried the *GALC* mutation. Analysis of the data from the first patient showed that the sequence data of the same *GALC* gene was missing because of insufficient sequencing depth. Cases 2 and 3 also had *NDUFAF1* gene mutations, which were also similarly filtered out during data analysis. Gene sequencing analysis was further performed on the healthy 5-year-old child in the pedigree, and the results showed heterozygous mutation in the *GALC* gene (c.599C>A (p.Ser200^*^) and compound heterozygous *NDUFAF1* mutations (c.278A>G; p. His93Arg heterozygous); c.247G>A; p.Asp83Asn wild type). In this family, three children simultaneously carried pathogenic mutations in the two genes, both of which are characterized by autosomal recessive inheritance. Thus, should we consider the mitochondrial gene *NDUFAF1* as co-disease-causing in children?

The *GALC* gene and the *NDUFAF1*gene are located at 14q31.31 and 15q15.1 (2, 3), respectively. To exclude the mutation of these two genes in the same chromosome region due to chromosomal translocation, chromosome karyotyping and FISH verification were performed to exclude the possibility that the mutation was caused by chromosomal translocation. Furthermore, we needed to examine whether these two gene mutations caused the corresponding dysfunction. We tested the enzyme activity of GALC and mitochondrial enzyme activity and verified them in 3 healthy individuals in the family.

The biochemical analysis in case 3 revealed a low GALC enzyme activity of 1.1 nmol/17 h/mg protein (normal >12.7 nmol/17 h/mg protein), confirming its deficiency. We identified the clinical phenotype of case 3 as infantile-onset (at 3 months) KD, characterized by progressive neurological deterioration in infancy, death before 6 months, and low GALC enzyme levels. GALC biochemical deficiency was not confirmed in cases 1 and 2, because they died. The healthy 5-year-old brother in the family has no symptoms and has been a healthy carrier thus far. He achieved normal developmental milestones, and no abnormal clinical manifestations were found. He had normal brain MRI results and GALC activity in a blood sample (48.19 nmol/17 h/mg protein).

**Figure 2:**
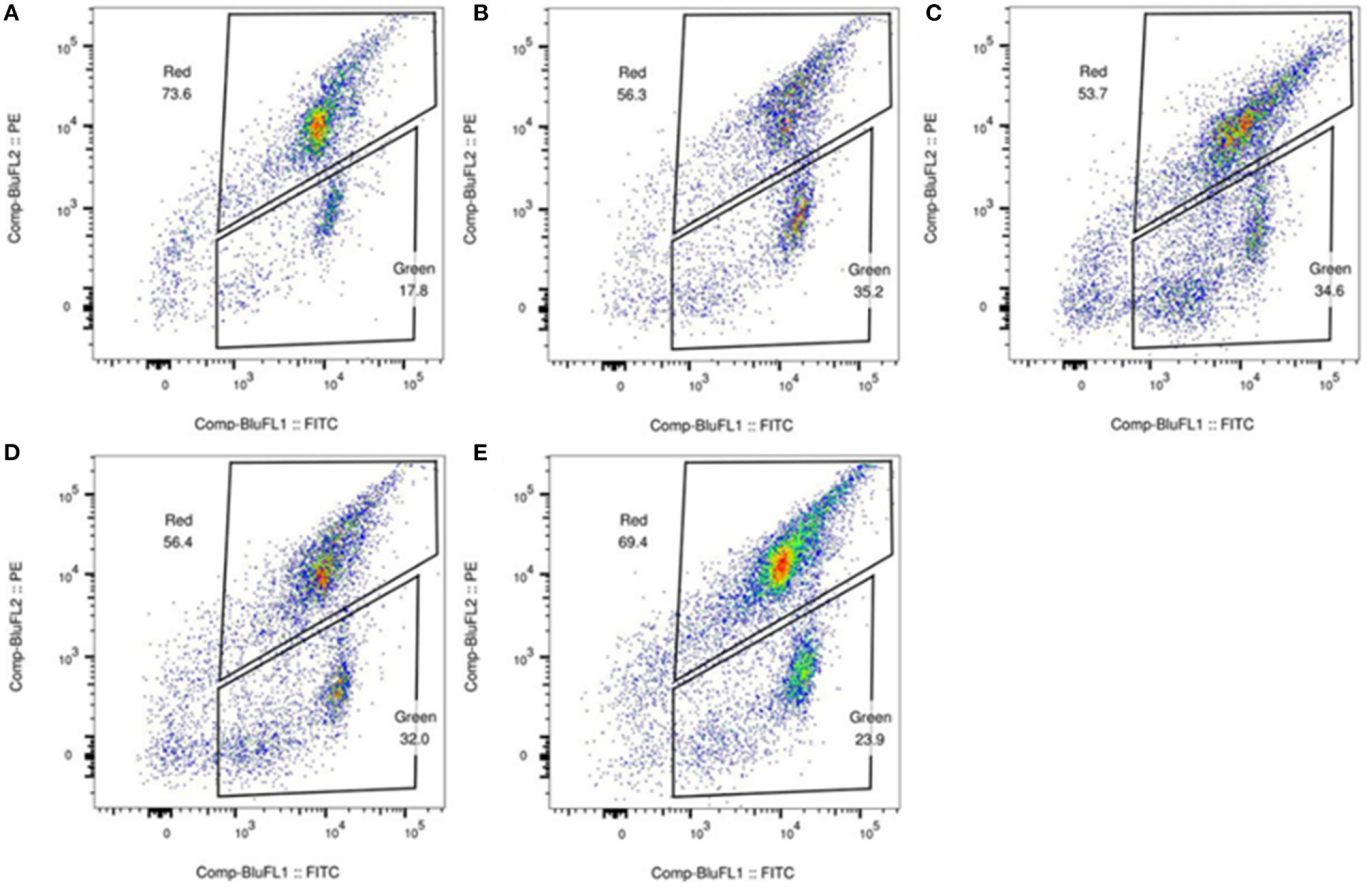
Detection of mitochondrial membrane potential in white blood cell. The flow cytometry analysis of the fresh peripheral blood white blood cell in the patients **(B)** (the father), **(C)** (case 3), **(D)** (the health 5-year-old child), **(E)** (the mother), and healthy control **(A)** stained with JC-1. The result revealed that the ratio of red to green fluorescence of **(B–E)** is lower than that of healthy group **(A)**, suggesting that the mitochondrial membrane potential of **(B–E)** is likely impaired.

JC-1 staining was performed to determine whether the mitochondrial membrane potential of the patients was decreased, and examine the mitochondrial function of the patients. JC-1 is an ideal fluorescent probe for the detection of mitochondrial membrane potential. When the mitochondrial membrane potential is high, JC-1 accumulates in the mitochondrial matrix and forms a polymer that produces red fluorescence; when the mitochondrial membrane potential is low, JC-1 cannot accumulate in the mitochondrial matrix, and it is a monomer that produces green fluorescence. Flow cytometry was performed to analyze the red-green fluorescence ratio of leukocytes stained with JC-1. The results of flow cytometry showed that the mitochondrial function in leukocytes was impaired (Figure 2).

## Discussion

Herein, we described a family with three children affected by hearing/visual impairment with infantile developmental delay and peripheral neuropathy with an autosomal recessive mode of inheritance. In order to verify whether the two genes cooperated to cause the disease, we examined the GALC enzymatic activity of case 3 and healthy children and parents, and the pathogenicity of *GALC* gene was confirmed by deficient galactocerebrosidase activity, and the synergism of mitochondrial gene was confirmed by flow cytometry and mitochondrial complex enzyme activity changes. We finally diagnosed the patients in this Chinese family with KD associated with mitochondrial dysfunction. The GALC/NDUFAF1 double gene mutations were confirmed, and can help in clinical diagnosis. In clinical practice, it is not infrequent to see variants in multiple genes simultaneously detected in a genetic report, and we tend to ignore genes whose phenotypes do not match to those of patients. It may be possible to detect a disease-causing gene without performing a deep analysis of other mutations.

There are two major phenotypes of KD: type 1 (infantile-onset, characterized by the first 6 months after birth, including irritability, retardation or regression in development, limb spasm, axial hypotonia, the disappearance of reflexes, optic atrophy, microcephaly, peripheral neuropathy, and death before the age of 2 years) and type 2 (late-onset, with slower disease progression that may even be detected during the adult phase) (2). Majority of patients present a deficiency of the GALC enzyme (4). Our patients met most of the diagnostic criteria for KD type 1, but the *NDUFAF1* mutations detected in the first patient sparked our interest in further exploration. Mutations in the *NDUFAF1* gene have been shown to be associated with human complex I deficiency. Studies have shown that NDUFAF1 is a vital mitochondrial protein that is involved in complex I assembly and stability. Mitochondrial complex I is the largest enzyme complex in the respiratory chain, and it has essential functions in electron transfer and proton pumping (5, 6). The clinical symptoms are identified with an early onset that is more likely to be associated with developmental delay, lactic acidosis, cardiomyopathy, and hypotonia (7). It seems to be similar to KD. Finally, we found that case 3 and the parents had changes in mitochondrial function after verification.

Decreased mitochondrial membrane potential was found in human oligodendrocytic cells in KD in 2014 (8). Other evidence of mitochondrial dysfunction in neuronal models was found, such as G_M1_-gangliosidosis, mucopolysaccharidosis IIIC, and Niemann Pick disease type C, which involve mitochondrial mass, morphology, and function (9). Although mitochondrial dysfunction has been confirmed in several lysosomal storage diseases, there are few data on the coexistence of mitochondrial gene mutations. In digenic inheritance, the mechanism of the co-pathogenesis of two genes is known to occur in various ways, such as intergene interaction, metabolic pathway interaction, and protein function interaction that can cause the disease phenotypes. To identify new therapeutic targets, the mechanisms of mitochondrial dysfunction in KD need to be further investigated.

In addition, the quality and data analysis of gene sequencing are vital and can easily lead to false-negative or false-positive results. With the extensive application of WES technology, we are bound to encounter some patients or families whose conditions cannot be explained by single-gene genetic diseases. Digenic inheritance also provides a new research idea for clinical diagnosis and scientific research. We report a family with *GALC* and *NDUFAF* gene co-pathogenesis, which we hope will provide new ideas for researchers.

## Conclusions

In this study, we identified a family with KD simultaneously carrying *NDUFAF1* mutations. Our study provides new insights into the understanding of the molecular pathogenesis of KD and mitochondrion-related genes, and further research is required to clarify the association between the observed clinical features and gene mutations. Families with pathogenic variants of the *NDUFAF1* and *GALC* genes may be identified in the future and should be analyzed comprehensively.

## Data Availability Statement

According to national legislation/guidelines, specifically the Administrative Regulations of the People's Republic of China on Human Genetic Resources (http://www.gov.cn/zhengce/content/2019-06/10/content_5398829.htm, http://english.www.gov.cn/policies/latest_releases/2019/06/10/content_281476708945462.htm), no additional raw data is available at this time. Data of this project can be accessed after an approval application to the China National Genebank (CNGB, https://db.cngb.org/cnsa/). Please refer to https://db.cngb.org/, or email: CNGBdb@cngb.org for detailed application guidance. The accession code CNP0002143 should be included in the application.

## Ethics Statement

The studies involving human participants were reviewed and approved by Institutional Review Board and Research Ethics Committee of the Hunan Children's Hospital, Changsha, Hunan (201603207). Written informed consent to participate in this study was provided by the participants' legal guardian/next of kin. Written informed consent was obtained from the individual(s), and minor(s)' legal guardian/next of kin, for the publication of any potentially identifiable images or data included in this article.

## Author Contributions

SG designed and organized the study. LW drafted the work and revised it critically for important intellectual content. XL provided and analyzed all the data. SY revised the manuscript. All the authors read and approved the final version of the manuscript.

## Funding

This work was supported by grants from the National Natural Science Foundation of China (Nos. 82171453, 81671297), Natural Science Foundation of Hunan Province (2021JJ30393), and Huxiang Youth Talent Support Program (2021RC3117).

## Conflict of Interest

The authors declare that the research was conducted in the absence of any commercial or financial relationships that could be construed as a potential conflict of interest.

## Publisher's Note

All claims expressed in this article are solely those of the authors and do not necessarily represent those of their affiliated organizations, or those of the publisher, the editors and the reviewers. Any product that may be evaluated in this article, or claim that may be made by its manufacturer, is not guaranteed or endorsed by the publisher.

## Abbreviations

KD: Krabbe disease
PSY: psychosine
MRI: magnetic resonance imaging
NCS: nerve conduction study
VEP: visual evoked potential
BAEP: brainstem auditory evoked potential
PICU: pediatric intensive care unit
WES: whole exome sequencing
ALT: alanine aminotransferase
AST: aspartate aminotransferase
AKP: alkaline phosphatase
EMG: electromyography.
